# Rational design of interfacial sp C─S─Zn hybridization in ZnS/graphdiyne for mercury vapor capture

**DOI:** 10.1126/sciadv.aef9068

**Published:** 2026-06-19

**Authors:** Chuanqi Pan, Cui Jie, Ziheng Xia, Kaiyue Xu, Yuan Yao, Honghu Li

**Affiliations:** ^1^ College of Environmental Engineering, Henan University of Technology, Zhengzhou 450001, P. R. China.; ^2^ Research Center for Environment and Health, School of Information Engineering, Zhongnan University of Economics and Law, Wuhan, Hubei 430073, P. R. China.; ^3^ Institute of Process Engineering, Chinese Academy of Sciences, Beijing 100190, P. R. China.

## Abstract

Capturing gaseous mercury (Hg^0^) from complex flue gases remains a common yet persistent issue. Here, the one-step hydrothermal method is adopted to synthesize zinc sulfide/graphdiyne (ZnS/GDY) nano adsorbents for efficient Hg vapor capture via a rational design of sp C─S─Zn hybridization on the interface. The research results indicate that GDY can gain electrons from ZnS though sp C─S─Zn bonds, which facilitates charge transfer and induces the generation of highly active Hg adsorption sites near sulfur defects (S_v_). The fabricated ZnS/GDY adsorbent can achieve a Hg^0^ removal efficiency of 98% and exhibits excellent resistance to 1500 parts per million high-concentration sulfur dioxide (SO_2_) constraints (maintaining a removal efficiency of 90.9%). Moreover, the commercial glass compound fiber filter (FMS) media coated with ZnS/GDY shows potential for application in bag filter systems. About 80% of Hg^0^ can be removed by ZnS/GDY@FMS, even under conditions of high SO_2_ concentration and in the presence of dust. Our work demonstrates that efficient Hg^0^ removal efficiency under high-concentration SO_2_ can be achieved by interfacial chemical bond engineering and provides guidance for functional carbon-based material design.

## Teaser

Interfacial chemical bond engineering enables the ZnS/graphdiyne for efficient mercury vapor removal under high-concentration SO_2_.

## INTRODUCTION

Mercury (Hg) is a common environmental pollutant and also the only heavy metal that can be fully cycled in ecosystems (*1*, *2*). Global anthropogenic Hg emissions continue to exceed 2100 tons per year (*3*). It mainly comes from coal and petroleum combustion, Hg and Au mining, metal smelting, and electrical manufacturing. Once Hg enters the atmosphere, it can be transported over long distances with the airflow and then settle into soil, leading to high levels of Hg in agricultural products. Once Hg reaches aquatic environments, it can be methylated into the biologically available toxic form of methylmercury (MeHg) in hypoxic environments (*4*, *5*). MeHg accumulates to a considerable concentration in fish and migrates to the human body along the food chain, causing Hg poisoning (Minamata disease) from MeHg, which poses a serious threat to the ecological environment and human health (*6*). Therefore, developing efficient and sustainable capture technologies and adsorbents for mercury are vital to public health and ecosystem protection.

Adsorption technology is one of the commonly used techniques for removing Hg vapor (*7*). Adsorbents such as activated carbon and molecular sieves have a large specific surface area and rich pore structure, and can efficiently capture gaseous elemental mercury (Hg^0^) through physical and chemical adsorption (*8*). In addition, the Hg^0^ vapor removal technology currently has great potential for development as it can work in environments with different concentrations of Hg vapor and generate minimal secondary pollution. Zinc sulfide (ZnS) exists in the form of sphalerite and wurtzite in nature, and is widely distributed (*9*). Its abundant reserves make it an important industrial raw material. ZnS is relatively cheap and suitable for large-scale industrial applications (*10*). However, the structure of ZnS is relatively stable, and its adsorption capacity for mercury has a certain limit (*11*, *12*). It can usually be doped and modified to further improve its mercury removal performance (*13*, *14*). The high specific surface area and rich π-electron structure of carbon-based materials (mainly sp^2^-hybridized) enable the adsorption and enrichment of Hg vapor through physical adsorption processes such as van der Waals forces and π-π interactions. In previous studies, traditional sp^2^-hybridized carbon materials such as activated carbon or biochar just serve as carriers, increasing the dispersion of active sulfides and providing a certain porosity to promote the migration and diffusion of Hg (*15*). The surface charge distribution of sp^2^-hybridized graphene is relatively uniform, possibly making it difficult to generate strong electronic interactions with the active components loaded on the surface. In actual flue gas emission environments, other gases such as SO_2_ and NO*_x_* are also present (*16*). These impurity gases may compete with Hg for adsorption sites or react with adsorbents, thereby affecting the stability of adsorbents and adsorption performance for Hg (*17*). Graphdiyne (GDY) is connected by a benzene ring and a ─C≡C─ triple bond, forming a stable and elastic two-dimensional structure (*18*, *19*). This unique bonding architecture endows GDY with excellent stability under high-temperature and harsh chemical environments (*20*). It is particularly noteworthy that unique acetylene bonds (sp-hybridized) of GDY can tune the electronic structure of metals (*21*–*24*), forming stable interface structure, which is expected to enhance the adsorption performance for Hg^0^ (*25*). However, the formation of new intrinsic active adsorption sites induced by interfacial chemical bonds and the mechanism of promoting efficient adsorption of Hg^0^ have not been reported and elucidated.

Here, via interface structure control strategy, we prepared sp C─S─Zn hybridization on the interface between GDY and ZnS nanoparticle with considerable Hg^0^ removal efficiency. The synthesized ZnS/GDY adsorbent can achieve a Hg^0^ removal efficiency of 98%, and still exhibited excellent tolerance at a high concentration of 1500 parts per million (ppm) SO_2,_ with a removal efficiency of over 90%. The comprehensive characterization and density functional theory (DFT) calculation analysis results indicated that GDY obtained electrons from ZnS through sp C─S─Zn hybrid bonds, which is beneficial for charge transfer and induction of higher activity Hg^0^ adsorption sites adjacent to S_v_ sites. However, the interaction between ZnS clusters and graphene (G) was a typical van der Waals interaction. Even with a twofold increase in ZnS clusters loading and a 1.7-fold rise in adsorbent dosage, the mercury removal efficiency of the ZnS/G composite only reached 76.5%. In addition, the commercial FMS media coated with ZnS/GDY showed potential for application in bag filter systems. Even under conditions of high SO_2_ concentration and in the presence of dust, ~80% of Hg^0^ removal efficiency can be achieved by ZnS/GDY@FMS. These findings indicated a promising strategy meeting the application under actual working condition by rational interfacial engineering and shed lights on the design of efficient adsorbents for Hg^0^.

## RESULTS

### Synthesis and structural characterization

**Fig. 1.**
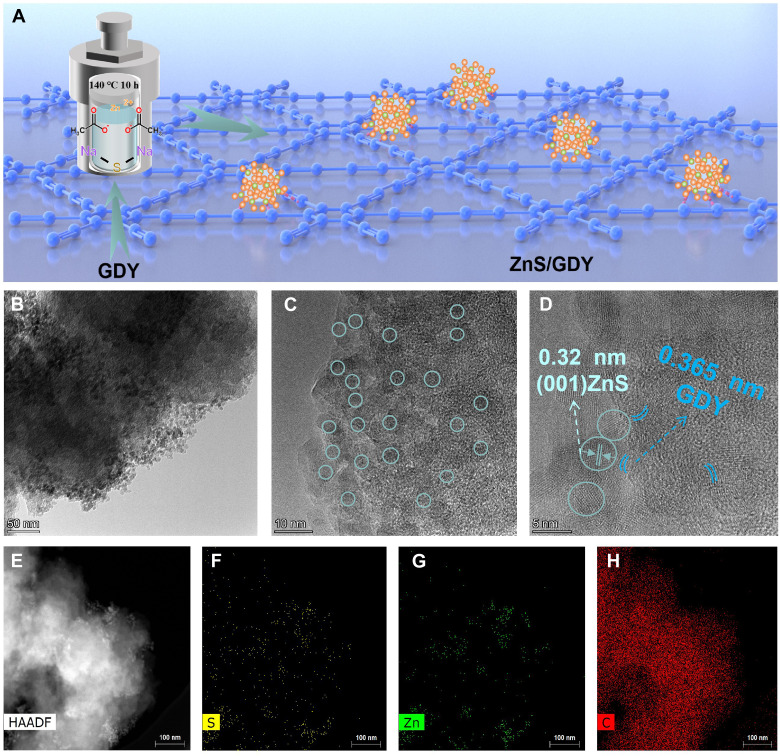
The morphology and electronic structure of ZnS/GDY. (**A**) The synthesis schematic diagram of ZnS/GDY composite material. (**B** to **D**) HR-TEM images of ZnS/GDY at different magnifications. (**E** to **H**) HAADF-STEM image and EDS mapping images (S, Zn, and C element) of ZnS/GDY.

The fabrication process of ZnS/GDY composite material is shown in Fig. 1A. The GDY was used as a support to construct ZnS/GDY composite nanomaterials through hydrothermal reactions 140°C for 10 hours. Last, the black powder sample was obtained by washing with deionized water and ethanol, and drying. The detailed synthesis process can be found in the experimental section. The microstructure and composition of ZnS/GDY were detected by high-resolution transmission electron microscopy (HR-TEM). As presented in Fig. 1 (B to D), we observed the morphology of ZnS/GDY in different regions and magnifications and found that ZnS nanoparticles were firmly anchored on GDY, with a particle size of approximately 5 nm. HR-TEM image of ZnS/GDY demonstrated the lattice fringes with an interplanar distance of 0.32 nm corresponds to the (001) plane of the wurtzite ZnS (*26*), the observed spacing of 0.365 nm belongs to the characteristic interlayer spacing of GDY (Fig. 1D) (*27*, *28*). Figure 1E clearly showed the high-angle annular dark-field scanning transmission electron microscopy (HAADF-STEM) images of ZnS/GDY. The dispersed bright dots represent the ZnS clusters on GDY, further confirming the successful anchoring of ZnS on GDY. Additional energy-dispersive x-ray spectroscopy mapping analysis revealed the dispersion of S, Zn, and C (Fig. 1, F to H), respectively. On the basis of the obtained HAADF-STEM images (Fig. 1E), the energy-dispersive x-ray spectroscopy (EDS) spectra were recorded as shown in fig. S1. The quantitative results of the EDS spectrum are summarized in table S1. These characterization and analytical results demonstrated the successful anchoring of uniformly dispersed ZnS clusters on GDY, confirming the superior capability of GDY’s unique triangular pores composed of alkyne-bonded carbon atoms in confining nanoclusters.

**Fig. 2.**
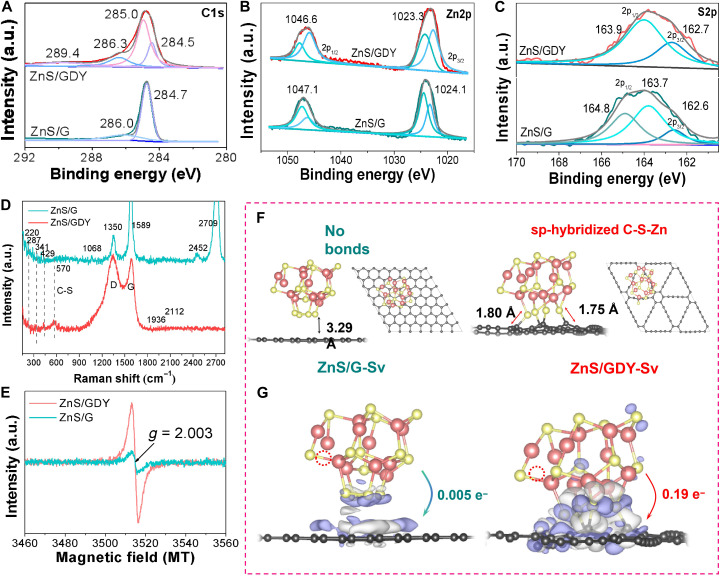
Physicochemical structure and theoretical model construction of ZnS/GDY. (**A**) The XPS C1s survey spectrum of ZnS/GDY and ZnS/G. (**B**) The XPS Zn2p survey spectrum of ZnS/GDY and ZnS/G. (**C**) The XPS S2p survey spectrum of ZnS/GDY and ZnS/G. (**D**) The Raman spectrum of ZnS/GDY and ZnS/G. (**E**) The EPR spectrum of ZnS/GDY and ZnS/G. (**F**) Top and side views of the geometric structures for the adsorption of the ZnS on G and GDY, the optimized molecular models of ZnS/G and ZnS/GDY based on DFT (The yellow, pink and black spheres refer to S, Zn, and C atoms, respectively). (**G**) The charge density difference of ZnS/G and ZnS/GDY, where electron accumulation and depletion are represented in gray and purple.

The composition and electronic structure of the synthesized sample ZnS/GDY were detected by the x-ray photoelectron spectroscopy (XPS). As shown in fig. S2, ZnS/GDY and ZnS/G are mainly composed of four elements: C, Zn, S, and O. Figure 2A shows the C1s spectrum of ZnS/GDY and ZnS/G. In the spectrum of ZnS/GDY, displays characteristic peaks with binding energy at 284.5, 285.0, 286.3, and 289.4 eV, attributed to C─C (sp^2^), C─C (sp), superposition of C─O and C─S bond, and C═O bonds, respectively (*29*). For ZnS/G, the characteristic peaks with binding energy at 284.7 and 286.0 eV were detected and attributed to C─C (sp^2^) and C─O (*30*), respectively. The XPS Zn2p region was also detected and presented in Fig. 2B. For ZnS/G, the binding energy at 1024.1 and 1047.1 eV were corresponded to the Zn 2p_3/2_ and Zn 2p_1/2_ of Zn^2+^ in the ZnS phase (*26*). Regarding the ZnS/GDY sample, the Zn 2p binding energy exhibited a shift toward lower binding energy, which can be attributed to the formation of chemical bonds between GDY and ZnS along with an associated electron transfer process. In addition, two additional peaks appeared at the binding energies of 1022.0 and 1045.0 eV, which may be due to the chemical bond formed at the interface inducing S defects (S_v_) in ZnS/GDY. The binding energies of S 2p for ZnS/GDY and ZnS/G were centered mainly at 162.6, 164.0, and 164.8 eV (Fig. 2C). These were assigned to S^2−^, S_2_^2−^, and S^0^ species, respectively (*31*). To further detect and confirm the surface electronic and compositional structure of ZnS anchored by GDY, Raman spectroscopy and electron paramagnetic resonance (EPR) were used to analyze their characteristic structures and verify the presence of S_v_. As depicted in Fig. 2D, the detected signals are mainly located in the wave number range of 200 to 1000 cm^−1^ and 1000 to 2800 cm^−1^. For ZnS/G, characteristic peaks at 220, 287, 341, and 429 cm^−1^ were detected, belonging to S^0^ and the characteristic peak of cubic ZnS, respectively (*32*). In addition, the D and G band peaks of G were also detected, located at 1350, 1589, and 2709 cm^−1^, respectively. For ZnS/GDY, in addition to the characteristic peak of ZnS, the C─S bond at 570 cm^−1^ was detected (*33*). Simultaneously, we observed a decrease in the strength of the diacetylene bond in GDY located at 1936 and 2112 cm^−1^ (*34*, *35*), further demonstrating that the diacetylene bond C atom in GDY forms C─S bond at the interface with the S atom in ZnS. EPR were performed to quantitatively determine the unpaired electrons, which have been widely used to describe the presence of vacancies. As presented in Fig. 2E, the ZnS/GDY exhibited a stronger EPR signal at the g factor of 2.003 compared to ZnS/G, further confirming the abundant S_v_ involved in the ZnS/GDY. (*36*–*38*)

On the basis of the above characterization techniques and analysis results, ZnS nanoclusters were truncated from the ZnS (001) crystal plane, and a stable configuration was obtained after geometric optimization. Subsequently, the optimized stable ZnS configuration was further optimized and matched with the GDY. The geometrical and electronic structure of ZnS/GDY was further investigated by placing a ZnS nanocluster with S_v_ on GDY via DFT calculations. The optimized stable structures of the ZnS/GDY were presented in Fig. 2F, showing that the ZnS cluster connected with GDY via interfacial C─S─Zn bonds and all C atoms are sp-hybridized in these bonds. However, G cannot form chemical bonds with ZnS. The distance between the ZnS cluster and graphene is in the range of ~3.29 Å, which is a typical van der Waals interaction range and consistent with literature reports (*39*, *40*). In addition, to better understand the electron transfer at the unique interface formed by sp-hybridized C atoms and the ZnS, the charge density difference was used to analyze the electron behavior. The charge distribution shows that GDY can gain electrons from ZnS, which facilitates charge transfer and induces the generation of highly active Hg adsorption sites (Fig. 2G). Estimated by Bader charge calculations, the GDY of a unit cell obtained 0.19 e^−^ from ZnS in total. For ZnS/G, only 0.0005 e^−^ electron transfer behavior occurred between ZnS and G, further indicating the strong interaction between GDY and ZnS through the unique C─S─Zn bond tuning.

### The Hg^0^ adsorption performance evaluation

**Fig. 3.**
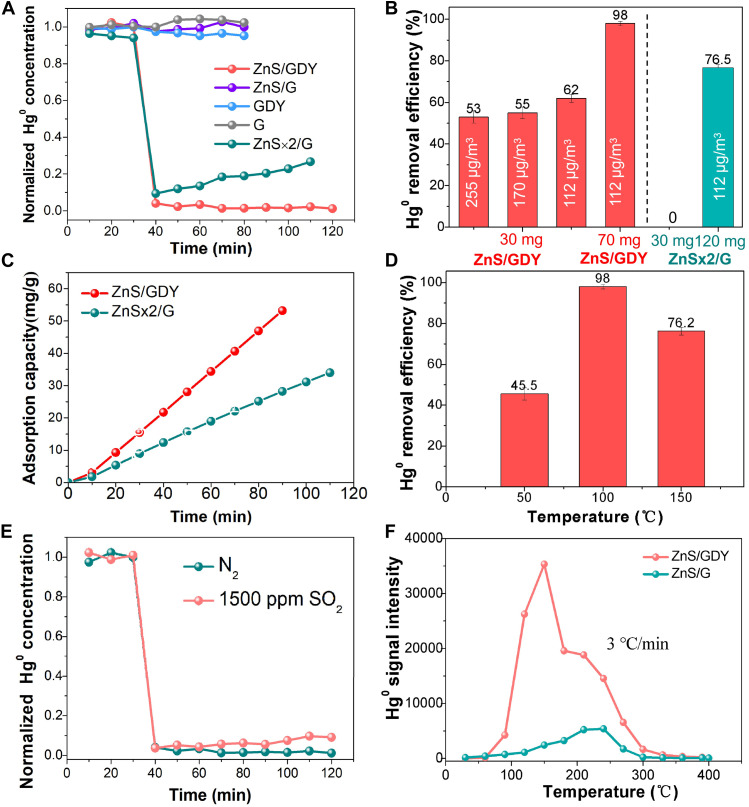
The Hg^0^ adsorption performance evaluation. (**A**) The Hg^0^ adsorption performances of different carbon sorbents (dash line indicates time point for gas flow passing through sorbent). (**B**) The removal efficiency of ZnS/GDY and ZnS/G under different adsorbent dosages and Hg concentrations. (**C**) The Hg adsorption capacity of ZnS/GDY (70 mg) and ZnS/G (120 mg, with a twofold increase in ZnS clusters loading). (**D**) The removal efficiency of ZnS/GDY at different temperature. (**E**) The Hg^0^ adsorption performances of ZnS/GDY under different working conditions Hg^0^ + N_2_ and Hg^0^ + SO_2_. (**F**) The Hg^0^ temperature programmed desorption of ZnS/GDY and ZnS/GDY.

The Hg^0^ removal performance of ZnS/GDY under different adsorbent dosages and Hg concentrations was evaluated in comparison with ZnS/G by the lab-scale fixed-bed reaction system (fig. S3). As shown in Fig. 3 (A and B), the ZnS/GDY can acquire a stable Hg^0^ removal efficiency of around 98% during the testing period, with a Hg^0^ concentration of 112 μg/m^3^ and an adsorbent dosage of only 70 mg. Even under high-concentration mercury-containing flue gas at 255 μg/m^3^, ZnS/GDY still achieves a Hg^0^ removal efficiency of 53%. However, ZnS/G was observed to be almost ineffective in Hg^0^ removal. ZnS supported on GDY can exert more prominent performance. The Hg^0^ removal performance of ZnS/G can be enhanced by increasing the amount of ZnS loading (twofold) and ZnS/G usage (120 mg) that can increase the active sulfur sites for Hg^0^ removal. Nevertheless, the removal efficiency of Hg^0^ by the ZnS/G adsorbent only increased from 0 to 76.5%. The Hg^0^ adsorption capacity of ZnS/GDY and ZnS/G has also been further calculated (Fig. 3C). After 100 min, the Hg adsorption capacity of ZnS/GDY reaches 54 μg/g, which was 1.8 times and 8 times higher than that of ZnS/G and pure GDY, respectively. Furthermore, the mercury adsorption capacity of the ZnS/GDY composite was systematically evaluated. As illustrated in fig. S4, the ZnS/GDY material achieved a high Hg adsorption capacity of 3001 μg·g^−1^ at 16% breakthrough. The effect of temperature on Hg^0^ removal over ZnS/GDY was examined as shown in Fig. 3D. When the temperature increases from 50° to 100°C, Hg^0^ removal efficiency increases from 45.5 to 98.0%. An increase in temperature can enhance molecular kinetic energy and accelerate the rate of reaction between Hg^0^ and active sulfur sites. With further increase of temperature to 150°C, the Hg^0^ removal efficiency displays a decreasing trend, which might be attributed to the desorption of adsorbed mercury species. Therefore, ZnS/GDY can achieve the highest Hg^0^ removal efficiency at 100°C. The specific surface area and pore size distribution of ZnS/GDY and ZnS/G were analyzed as shown in fig. S5, and the specific test results and analysis are as follows: The specific surface areas of ZnS/GDY (32.6 m^2^/g) and ZnS/G (30.5 m^2^/g) are quite similar, showing no significant difference. In addition, pore size distribution analysis indicates that both samples exhibit typical mesoporous material characteristics, with no significant difference in pore structure type. These results indicate that the efficient Hg removal performance and stability of ZnS/GDY are attributed to the C─S─Zn hybrid bonds formed at its interface and the induced active Hg adsorption sites. Hg^0^ associated with sulfur is widely present in fossil fuels and mineral resources, and its combustion or smelting process produces SO_2_, which is several orders of magnitude higher in concentration than Hg^0^ and has a strong impact on the Hg^0^ removal performance of catalysts/adsorbents. Therefore, the effect of SO_2_ on Hg^0^ removal over ZnS/GDY was further investigated as shown in Fig. 3E. It can be seen that ZnS/GDY can still achieve about 90.9% removal under 1500 ppm SO_2_, which suggested that ZnS/GDY has excellent SO_2_ resistance. In addition, we further evaluated the adsorption performance of ZnS/GDY for Hg^0^ in a 400 ppm NO atmosphere. The ZnS/GDY can still achieve about 93% removal efficiency under 400 ppm NO, which suggests that ZnS/GDY has also excellent NO resistance (fig. S6). The temperature programmed desorption of Hg (Hg-TPD) was further studied to illuminate the mercury existence over Hg-laden ZnS/GDY. As depicted in Fig. 3F, the Hg-TPD profile for ZnS/GDY shows a desorption peak at 150°C with high intensity and a shoulder peak at 210°C, which can be attributed to weakly adsorbed mercury and HgS (*41*). Last, to further investigate the regeneration performance of the ZnS/GDY composite in the presence of 800 ppm SO_2_, cyclic regeneration experiments were performed. As illustrated in fig. S7, the ZnS/GDY material maintained outstanding Hg removal efficiency even after five consecutive regeneration cycles. This remarkable performance confirms that the as-prepared ZnS/GDY has excellent reusability and practical application potential in flue gas mercury removal.

### The Hg^0^ adsorption mechanism over ZnS/GDY

**Fig. 4.**
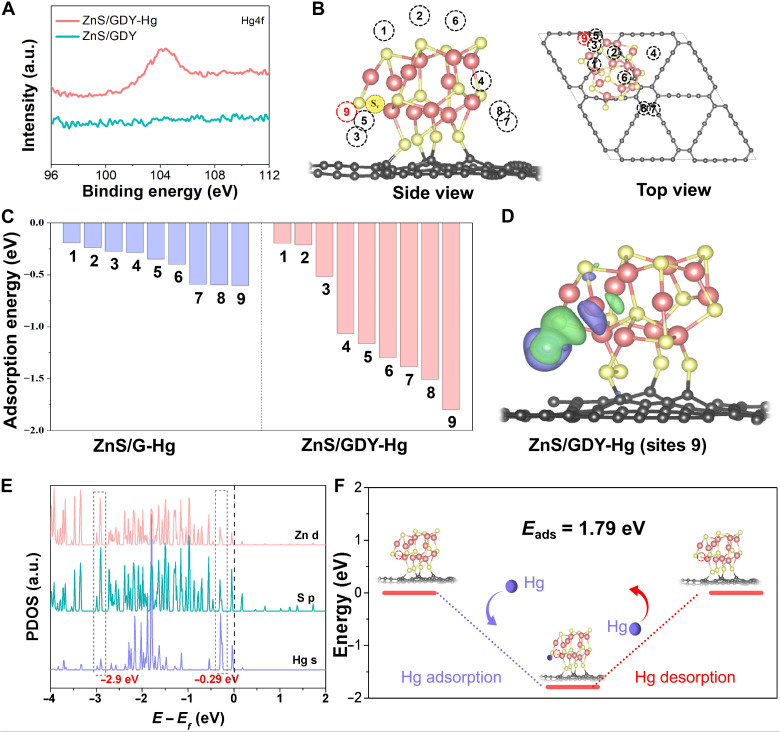
The Hg^0^ adsorption mechanism over ZnS/GDY. (**A**) The XPS Hg 4f spectrum of ZnS/GDY-Hg and bare ZnS/GDY. (**B**) Top and side views of the geometric structures for different adsorption sites of the Hg on ZnS/GDY (the yellow, pink and black spheres refer to S, Zn, and C atoms, respectively). (**C**) The calculation of adsorption energy of Hg atoms at different sites on the ZnS/G and ZnS/GDY optimized models. (**D**) The charge density difference of ZnS/GDY-Hg, where electron accumulation and depletion are represented in purple and green. (**E**) The PDOS comparison for Zn d, S p, and Hg s orbitals within ZnS/GDY-Hg. The Fermi level is set to zero. (**F**) Adsorption and desorption energy calculation of Hg atom on ZnS/GDY. Insets show the side views of a snapshot of the atomic configuration.

To clarify the efficient Hg adsorption performance of the ZnS/GDY adsorbent, the possible adsorption sites and interaction mechanisms of ZnS/GDY for Hg atom was extensively explored. The XPS Hg 4f spectrum of ZnS/GDY after Hg adsorption was performed. As shown in Fig. 4A, the characteristic peak of 104 eV ascribed to Hg is detected in the ZnS/GDY sample (*15*). First, we have calculated the possible adsorption sites and adsorption energies of Hg atoms on the GDY surface. The possible adsorption configurations and adsorption energies of the Hg atom on GDY at different sites are displayed in table S2. It can be seen that the calculated maximum adsorption energies of Hg onto GDY for the optimal adsorption configuration is only −0.24 eV, indicating that GDY has relatively poor adsorption capacity for Hg. Furthermore, nine potential adsorption sites of Hg atoms on the ZnS/GDY (Fig. 4B) and ZnS/G were comprehensively investigated (fig. S8). The adsorption energies of Hg atom at different sites on ZnS/GDY and ZnS/G were calculated and summarized in tables S3 and S4. The adsorption energies of Hg atoms at different sites on ZnS/GDY and ZnS/G are summarized in Fig. 4C. It was clearly observed that the adsorption energies of Hg atom on ZnS/GDY are generally much higher than those on ZnS/G. Notably, site 9 adjacent to the S_v_ is most conducive to the adsorption of Hg atoms and had a higher adsorption energy. Specifically, for ZnS/GDY, the adsorption energy of Hg atoms at site 9, which is directly adjacent to the S_v_, reaches −1.79 eV, while the adsorption energy of Hg atoms on ZnS/G was only −0.6 eV, indicating that the unique C─S─Zn hybrid bonds formed at the interface between the acetylenic C in GDY and ZnS induce the formation of high active Hg atom adsorption S sites adjacent to the S_v_. These demonstrated features are beneficial for the effective removal of Hg. To confirm and reveal the electronic interaction between Hg atoms and active S sites, Bader charge calculations were performed (Fig. 4D). It can clearly observe that electron accumulation and depletion mainly occur on Hg and S atoms adjacent to Sv, confirming that the S site was a real active site that can effectively adsorb Hg atoms in the form of chemical adsorption. Figure 4E presents the PODS of ZnS/GDY after adsorbing Hg atom. The remarkable hybridization peaks between Hg-s and S-p orbitals around −2.9 and −0.26 eV below the Femi level further confirm the relatively strong orbital coupling between Hg and S atom adjacent to S_v_ (*42*, *43*), which endow ZnS/GDY adsorbent an excellent adsorption performance for Hg. In environmental governance or industrial applications, it is required that adsorbents have irreversible or difficult to desorb Hg^0^ adsorption to prevent Hg^0^ from being re-released into the environment in subsequent processes, causing secondary pollution and achieving effective Hg^0^ pollution control. Subsequently, we calculated the adsorption and desorption energies of Hg atom on the ZnS/GDY. As shown in Fig. 4F, Hg atoms diffused and were then adsorbed onto the S site adjacent to Sv of ZnS/GDY has a chemical adsorption energy of −1.79 eV. Therefore, the desorption of Hg atoms from the ZnS/GDY adsorbent theoretically requires overcoming an energy barrier as high as 1.79 eV. These characterizations and theoretical analysis results collectively demonstrated that the C─S─Zn hybridized bonds at the interface of the ZnS/GDY composite nanomaterials induce the formation of more active S sites adjacent to S_v_, thereby enhancing the efficient adsorption capacity for Hg.

### The Hg^0^ capture by ZnS/GDY modified filter mediums in complex flue gas

**Fig. 5.**
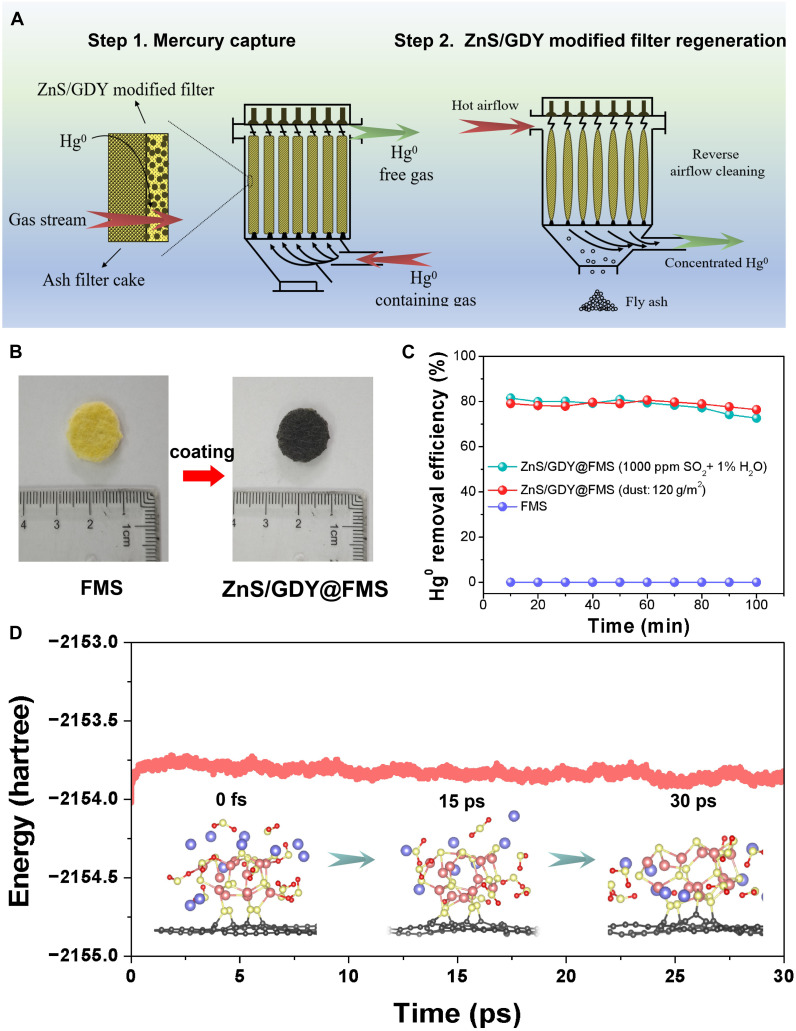
The Hg^0^ capture by ZnS/GDY modified filter mediums in complex flue gas. (**A**) The schematic diagram of the improved bag filter (ZnS/GDY modified filter mediums) systems for Hg^0^ capture. (**B**) The macroscopic images of FMS and ZnS/GDY@FMS. (**C**) The Hg^0^ removal efficiency of FMS and ZnS/GDY modified filter mediums in different working conditions. (**D**) Molecular dynamic simulation of SO_2_ + Hg over ZnS/GDY at 150°C. The whole reaction process is uncovered by displaying the snapshots of critical structures at 0, 15, and 30 ps in order (the yellow, pink, black, red, and purple spheres represent S, Zn, C, O, and Hg atoms, respectively).

On the basis of the outstanding Hg^0^ capture performance of ZnS/GDY, an improved fabric filter system has been proposed, which can effectively capture Hg^0^ from flue gas. The Hg^0^ capture by ZnS/GDY can be integrated with the dust removal systems currently equipped in coal-fired power plants, further extending its application under actual operation conditions. As shown in Fig. 5A, the filtration units for Hg^0^ capture can be connected to the integrated fabric filter through a zoned design. The filter media can be coated with ZnS/GDY to subsequently immobilize Hg^0^ from the flue gas. Fly ash can be removed from the filter media via reverse air flow (hot) cleaning. Meanwhile, Hg^0^ species will undergo desorption, and the concentrated Hg^0^ can be recovered through condensation for industrial use. Such technical route requires the filter media to have good high-temperature resistance. Therefore, a typical commercial filter medium, namely glass compound fiber filter material (FMS), was selected and evaluated as a blank control for its Hg^0^ adsorption performance. The coating procedure was briefly described as follows: First, ZnS/GDY was dispersed in deionized water via ultrasonication to form a suspension. Subsequently, ZnS/GDY was deposited onto the filter medium by vacuum filtration. Figure 5B displays photographs of both FMS and ZnS/GDY@FMS before and after coating. And the scanning electron microscopy images of FMS and ZnS/GDY@FMS are displayed in the fig. S9. Then, the simulated flue gas containing 75 μg/m^3^, 1000 ppm SO_2_, 1% vol. H_2_O, balanced N_2_ and 75 μg/m^3^, 120 g/m^2^ dust, and balanced N_2_ passed through the filter mediums and the outlet Hg^0^ concentration was detected. The Hg^0^ removal efficiency of ZnS/GDY@FMS filter mediums were displayed in Fig. 5C. It can be seen that the pristine FMS showed no Hg^0^ capture capability. However, ZnS/GDY@FMS exhibit excellent Hg^0^ removal abilities. About 80% of Hg^0^ can be removed by ZnS/GDY@FMS, and even under conditions of high SO_2_ concentration and in the presence of dust, its Hg^0^ removal efficiency exhibited only a modest decrease. These findings demonstrated that the Hg^0^ removal capability of the filter medium was substantially improved upon the incorporation of ZnS/GDY. This suggested the potential for integrating ZnS/GDY-based Hg^0^ capture with established dust removal systems. Further molecular dynamic (MD) simulation based on first principle at 100° and 150°C reproduces the effect of SO_2_ molecules on the competitive adsorption of active sites and sp C─S─Zn hybrid bond structure clearly in real time. The adsorption energy calculation of single SO_2_ molecules at the active S site adjacent to S_v_ was performed. The calculation results show that its adsorption energy was only −0.43 eV (fig. S10), much lower than the adsorption energy of Hg atoms at the active S site (−1.79 eV), which rules out the possibility of competitive adsorption between SO_2_ molecules and Hg atoms.

In addition, we conducted MD simulations to investigate the effects of SO_2_ atmosphere on the ZnS/GDY structure at actual reaction temperatures, with particular focus on the stability of the sp-hybridized C─S─Zn bonds at the interface. As shown in Fig. 5D and fig. S11, the critical configurations of key steps were displayed in snapshots of 0, 15, and 30 ps to track and visualize the whole procedure. It can be clearly observed that the GDY anchored ZnS nanocluster structure exhibits exceptional stability under SO_2_ + Hg atmosphere. The interfacial C─S─Zn hybrid bonds exhibit favorable stability at 150°C. Although a certain degree of thermal vibration exists, the bonding structure remains essentially unchanged throughout the simulation. The simulation results revealed that the excellent stability of ZnS/GDY structure through the formation of C─S─Zn bonds at the interface was responsible for the high Hg adsorption performance in a high-concentration SO_2_-mixed atmosphere. Different from G with sp^2^ hybridization carbon, sp-hybridization containing carbon material GDY exhibits unique features (*44*–*48*). The sp-hybridized carbon atoms, which bonded with S atoms, can extraordinarily enhance Hg adsorption performance due to promote interfacial electron transfer through C─S─Zn bonds and induce the generation of highly active Hg adsorption sites near sulfur defects.

## DISCUSSION

In conclusion, the “sp C─S─Zn hybridization” constructed for the first time through interface engineering between ZnS clusters and GDY interface achieved excellent Hg^0^ removal efficiency. The behavior of ZnS clusters donating electrons was rapidly transferred to GDY through C─S─Zn hybrid bonds, inducing the generation of S sites adjacent to S_v_ with higher adsorption activity (−1.79 eV, compared to ZnS/G −0.60 eV), rather than S_v_ sites. The fabricated ZnS/GDY nano-adsorbent can achieve a Hg^0^ removal efficiency of 98.0%, and still exhibits excellent tolerance at a high concentration of 1500 ppm SO_2_ and NO, with a removal efficiency of over 90%. Even with a twofold increase in ZnS clusters loading and a 1.7-fold rise in adsorbent dosage, the Hg^0^ removal efficiency of the ZnS/G composite only reached 76.5%, which is mainly due to the typical van der Waals interaction between ZnS clusters and G. In addition, the ZnS/GDY material maintained outstanding Hg removal efficiency even after five consecutive regeneration cycles in a SO_2_ atmosphere. Furthermore, the ZnS/GDY-coated commercial FMS media represented a promising candidate for bag filter systems, capable of achieving approximately 80% Hg^0^ removal efficiency while retaining performance under high SO_2_ and dust conditions. This work demonstrated that efficient Hg^0^ removal efficiency can be achieved through interface chemical bonding engineering, even under complex operating conditions, and provides guidance for the design of functional composite carbon-based nanomaterials.

## MATERIALS AND METHODS

### Synthesis of the ZnS/GDY and ZnS/G

The synthesis of GDY method has been described in previous work (*20*). GDY (150 mg) and deionized water (30 ml) were mixed with sonication to a uniform homogeneous solution, and then 0.15 mmol of Zn (CH_3_COO)_2_·2H_2_O was added to the above solution with ultrasound for 1 hour (solution a). Na_2_S·9H_2_O (0.15 mmol) was dissolved into 20 ml of deionized water (solution b). Solutions a and b were mixed and stirred magnetically for 1 hour. The mixed solution was transferred to 100-ml Teflon reactor and heated under 140°C for 10 hours. The Teflon reactor naturally drops to room temperature, the obtained materials were washed several times with C_2_H_5_OH and water and were dried. The synthesis of Zinc sulfide/graphene (ZnS/G) is consistent with the above synthesis steps.

### Calculation

DFT was used to carry out first-principles calculations by using the Vienna ab initio simulation package (VASP) (*49*, *50*). In structural relaxation, exchange and correlation effects were calculated via the Perdew-Burke-Ernzerhof functional. The self-consistency accuracy was set as 10 to 6 eV and the force convergence was set as 0.02 eV·Å^−1^, and the plane wave energy cutoff was set as 400 eV. K-points were sampled in the Monhorst-Pack grid for the first Brillouin zone integration (*51*, *52*). The vacuum layer with 15 Å was set to prevent mirror interactions. All atoms were relaxed in the structural relaxation and simulations were dispersion-corrected by DFT-D3 to compensate for the calculation error of weak interactions (*53*, *54*). The VASPKIT software (version 1.5.1) was used to analyze wave function files (*55*). Ab initio MD calculations were carried out using the CP2K code (*56*). All calculations used a mixed Gaussian and plane wave basis sets.

## References

[1] P. J. Blanchfield, J. W. M. Rudd, L. E. Hrenchuk, M. Amyot, C. L. Babiarz, K. G. Beaty, R. A. D. Bodaly, B. A. Branfireun, C. C. Gilmour, J. A. Graydon, B. D. Hall, R. C. Harris, A. Heyes, H. Hintelmann, J. P. Hurley, C. A. Kelly, D. P. Krabbenhoft, S. E. Lindberg, R. P. Mason, M. J. Paterson, C. L. Podemski, K. A. Sandilands, G. R. Southworth, V. L. St Louis, L. S. Tate, M. T. Tate, Experimental evidence for recovery of mercury-contaminated fish populations. Nature 601, 74–78 (2022). 34912113 10.1038/s41586-021-04222-7 PMC8732272

[2] Y.-T. Wang, Z.-H. Li, S.-L. Gong, J. Jiang, Y.-L. Jiang, Y. Guan, Z. Wu, G. Liu, Y.-C. Tian, L.-J. Tian, Altering the biotransformation fate of mercury by coordinating respiratory, electron flow, and trapping module. Environ. Sci. Technol. 59, 19921–19931 (2025). 40932093 10.1021/acs.est.5c08193

[3] H. Li, F. Meng, P. Zhu, H. Zu, Z. Yang, W. Qu, J. Yang, Biomimetic mercury immobilization by selenium functionalized polyphenylene sulfide fabric. Nat. Commun. 15, 1292 (2024). 38346957 10.1038/s41467-024-45486-7 PMC10861514

[4] Q. Hong, H. Xu, X. Sun, J. Li, W. Huang, Z. Qu, L. Zhang, N. Yan, In-situ low-temperature sulfur CVD on metal sulfides with SO_2_ to realize self-sustained adsorption of mercury. Nat. Commun. 15, 3362 (2024). 38637534 10.1038/s41467-024-47725-3 PMC11026451

[5] M. Wu, X. Wu, A. S. Lopez, P. J. Blanchfield, H. Ren, H. Zhong, Climate change amplifies neurotoxic methylmercury threat to Asian fish consumers. Proc. Natl. Acad. Sci. U.S.A. 122, e2421921122 (2025). 40127279 10.1073/pnas.2421921122 PMC12002180

[6] Y. Xiang, G. Liu, Y. Yin, Y. Li, D. Wang, Y. Cai, G. Jiang, Human activities shape important geographic differences in fish mercury concentration levels. Nat. Food 5, 836–845 (2024). 39327525 10.1038/s43016-024-01049-z

[7] H. Zhang, T. Wang, Y. Zhang, J. Wang, B. Sun, W. Pan, A review on adsorbent/catalyst application for mercury removal in flue gas: Effect of sulphur oxides (SO_2_, SO_3_). J. Clean. Prod. 276, 124220 (2020).

[8] X. Zhang, X. Han, C. Gao, X. Wang, Y. Wei, N. Zhang, J. Bao, N. Xu, G. He, In-situ growth of Co/Zn bimetallic MOF on GO surface to prepare GO supporting Co@C single-atom catalyst for Hg^0^ oxidation. Fuel 333, 126135 (2023).

[9] S. Saleem, S. Khalid, M. A. Malik, A. Nazir, Review and outlook of zinc sulfide nanostructures for supercapacitors. Energy Fuels 38, 9153–9185 (2024).

[10] L. Chang, J. Li, Q. Sun, X. Liu, X. Lu, H. Cheng, Innovative zinc anodes: Advancing metallurgy methods to battery applications. Small 20, e2408124 (2024). 39428824 10.1002/smll.202408124

[11] X. Zheng, Y. Song, Q. Gao, J. Lin, J. Zhai, Z. Shao, J. Li, D. Wu, X. Shi, W. Liu, X. Tian, Y. Liu, Controllable-photocorrosion balance endows ZnCdS stable photocatalytic hydrogen evolution. Adv. Funct. Mater. 35, 2506159 (2025).

[12] W. Liu, H. Xu, Y. Guo, Y. Yuan, Y. Liao, Z. Qu, N. Yan, Immobilization of elemental mercury in non-ferrous metal smelting gas using ZnSe_1-x_S_x_ nanoparticles. Fuel 254, 115641 (2019).

[13] Y. Xiao, Y. Huang, H. Cheng, J. Wu, B. Jin, Development of copper sulfide functionalized CeO_2_ nanoparticle for strengthened removal of gaseous elemental mercury from flue gas. Chem. Eng. J. 453, 139773 (2023).

[14] J. Wang, J. Mei, C. Wang, Q. Hu, X. Zhang, S. Yang, Outstanding performance of ZnS/TiO_2_ for the urgent disposal of liquid mercury leakage indoors: Novel support effect, reaction mechanism and kinetics. J. Hazard. Mater. 403, 123867 (2021). 33264940 10.1016/j.jhazmat.2020.123867

[15] H. Li, C. Pan, X. Peng, B. Zhang, S. Song, Z. Xu, X. Qiu, Y. Liu, J. Wang, Y. Guo, In-situ adsorption-coupled-oxidation enabled mercury vapor capture over sp-hybridized graphdiyne. Nat. Commun. 16, 2439 (2025). 40069183 10.1038/s41467-025-57197-8 PMC11897331

[16] H. Zhang, Z. Li, T. Liu, M. Zhang, S. Deng, Y. Li, P. Liang, Satisfactory anti-interference and high performance of the 1Co-1Ce/Mn@ZSM-5 catalyst for simultaneous removal of NO and Hg^0^ in abominable flue gas. Environ. Sci. Technol. 56, 3596–3603 (2022). 35195995 10.1021/acs.est.2c00143

[17] H. Li, L. Zhu, J. Wang, L. Li, P. Lee, Y. Feng, K. Shih, Effect of nitrogen oxides on elemental mercury removal by nanosized mineral sulfide. Environ. Sci. Technol. 51, 8530–8536 (2017). 28662579 10.1021/acs.est.7b00224

[18] C. Pan, Q. He, C. Li, Promising graphdiyne-based nanomaterials for environmental pollutant control. Sci. China Mater. 67, 3456–3467 (2024).

[19] X. Zheng, S. Chen, J. Li, H. Wu, C. Zhang, D. Zhang, X. Chen, Y. Gao, F. He, L. Hui, H. Liu, T. Jiu, N. Wang, G. Li, J. Xu, Y. Xue, C. Huang, C. Chen, Y. Guo, T.-B. Lu, D. Wang, L. Mao, J. Zhang, Y. Zhang, L. Chi, W. Guo, X.-H. Bu, H. Zhang, L. Dai, Y. Zhao, Y. Li, Two-dimensional carbon graphdiyne: Advances in fundamental and application research. ACS Nano 17, 14309–14346 (2023). 37471703 10.1021/acsnano.3c03849

[20] C. Pan, C. Wang, X. Zhao, P. Xu, F. Mao, J. Yang, Y. Zhu, R. Yu, S. Xiao, Y. Fang, H. Deng, Z. Luo, J. Wu, J. Li, S. Liu, S. Xiao, L. Zhang, Y. Guo, Neighboring sp-hybridized carbon participated molecular oxygen activation on the interface of sub-nanocluster CuO/Graphdiyne. J. Am. Chem. Soc. 144, 4942–4951 (2022). 35262357 10.1021/jacs.1c12772

[21] X. Yin, H. Wang, S. Tang, X. Lu, M. Shu, R. Si, T. Lu, Engineering the coordination environment of single-atom platinum anchored on graphdiyne for optimizing electrocatalytic hydrogen evolution. Angew. Chem. Int. Ed. Engl. 57, 9382–9386 (2018). 29885267 10.1002/anie.201804817

[22] Z. Zheng, L. Qi, X. Luan, S. Zhao, Y. Xue, Y. Li, Growing highly ordered Pt and Mn bimetallic single atomic layers over graphdiyne. Nat. Commun. 15, 7331 (2024). 39187493 10.1038/s41467-024-51687-x PMC11347568

[23] H. Zou, W. Rong, S. Wei, Y. Ji, L. Duan, Regulating kinetics and thermodynamics of electrochemical nitrogen reduction with metal single-atom catalysts in a pressurized electrolyser. Proc. Natl. Acad. Sci. U.S.A. 117, 29462–29468 (2020). 33172992 10.1073/pnas.2015108117 PMC7703585

[24] Y. Gao, Y. Xue, L. Qi, C. Xing, X. Zheng, F. He, Y. Li, Rhodium nanocrystals on porous graphdiyne for electrocatalytic hydrogen evolution from saline water. Nat. Commun. 13, 5227 (2022). 36064713 10.1038/s41467-022-32937-2 PMC9445080

[25] S. Xie, C. Pan, Y. Yao, X. Yu, X. Ze, W. Yuan, Y. Zhang, N. Guo, X. Li, X. Mao, S. Xia, J. Li, Y. Guo, Ultra-high-efficiency capture of lead ions over acetylenic bond-rich graphdiyne adsorbent in aqueous solution. Proc. Natl. Acad. Sci. U.S.A. 120, e2221002120 (2023). 37036993 10.1073/pnas.2221002120 PMC10120024

[26] J. Zhao, X. Liu, X. Ren, X. Sun, D. Tian, Q. Wei, D. Wu, Defect-rich ZnS nanoparticles supported on reduced graphene oxide for high-efficiency ambient N_2_-to-NH_3_ conversion. Appl. Catal. B Environ. 284, 119746 (2021).

[27] X. Gao, J. Li, R. Du, J. Zhou, M. Huang, R. Liu, J. Li, Z. Wu, Z. Liu, J. Zhang, Direct synthesis of graphdiyne nanowalls on arbitrary substrates and its application for photoelectrochemical water splitting cell. Adv. Mater. 29, 1605308 (2017). 10.1002/adma.201605308 28009465

[28] H. Yu, Y. Xue, L. Hui, C. Zhang, Y. Fang, Y. Liu, X. Chen, D. Zhang, B. Huang, Y. Li, Graphdiyne-based metal atomic catalysts for synthesizing ammonia. Natl. Sci. Rev. 8, nwaa213 (2021). 34691704 10.1093/nsr/nwaa213 PMC8363333

[29] X. Luan, L. Qi, Z. Zheng, Y. Gao, Y. Xue, Y. Li, Step by step induced growth of zinc-metal interface on graphdiyne for aqueous zinc-ion batteries. Angew. Chem. Int. Ed. Engl. 62, e202215968 (2023). 36593176 10.1002/anie.202215968

[30] W. Zhang, M. Makurat, X. Liu, B. Chen, Y. Li, X. Liu, T. J. F. Kock, A. Jiao, G. Jiang, C. Leist, C. Maheu, H. Sezen, D. Calvani, I. Eren, L. Jiang, F. Buda, H. Qi, J. P. Hofmann, X. Feng, U. Kaiser, L. Sun, Z. Liu, A. Kuc, T. Heine, G. F. Schneider, Sulfophenylated centimeter-sized graphene membrane in a direct methanol fuel cell. Nat. Commun. 16, 10857 (2025). 41309551 10.1038/s41467-025-65507-3 PMC12675496

[31] L. Guan, Z. Chen, Y. Liu, R. Wang, K. Yan, Z. Xu, J. Li, Z. Liu, J. Li, H. Liu, Engineering sulfur-rich MoS_2_ adsorbent with abundant unsaturated coordination sulfur sites for gaseous mercury capture from high-concentration SO_2_ smelting flue gas. Chem. Eng. J. 483, 149122 (2024).

[32] J. Lin, J. Lei, Y. Zuo, Y. Lu, Y. Yan, P. Lin, R. Gao, M. Liu, W. Yan, J. Zhang, Electron bridge effect induced by iodide aqueous zinc-sulfur batteries. ACS Energy Lett. 10, 6456–6465 (2025).

[33] Q. Su, W. Wang, J. Chen, J. Ji, W. Wang, W. Ren, L. Zhang, J. Xie, Q. An, The out-of-plane C─S bonds boosting reversible redox in copper sulfide cathodes for ultradurable magnesium battery. Adv. Funct. Mater. 35, 2419594 (2025).

[34] Q. Bai, M. Liang, W. Wu, C. Zhang, X. Li, M. Liu, D. Yang, W. W. Yu, Q. Hu, L. Wang, F. Du, N. Sui, Z. Zhu, Plasmonic nanozyme of graphdiyne nanowalls wrapped hollow copper sulfide nanocubes for rapid bacteria-killing. Adv. Funct. Mater. 32, 2112683 (2022).

[35] J. Li, H. Cao, Q. Wang, H. Zhang, Q. Liu, C. Chen, Z. Shi, G. Li, Y. Kong, Y. Cai, Y. Wu, Z. Lai, Y. Han, J. Zhang, Space-confined synthesis of monolayer graphdiyne in MXene interlayer. Adv. Mater. 36, e2308429 (2024). 37865868 10.1002/adma.202308429

[36] X. Gao, L. Li, Z. Zhao, Y. J. Dappe, Z. Jiang, P. Song, Y. Wang, J. Zhu, Sulfur vacancy-rich ZnS on ordered microporous carbon frameworks for efficient photocatalytic CO_2_ reduction. Appl. Catal. B Environ. Energy 364, 124835 (2025).

[37] C. Yuan, H. Yin, J. Li, Y. Zhang, H. Chen, D. Xiao, Q. Wang, Y. Zhang, Q. Xue, Light-induced CoO_x_ surface reconstruction in hollow heterostructure for durable photocatalytic seawater splitting. Nat. Commun. 16, 6607 (2025). 40675963 10.1038/s41467-025-62033-0 PMC12271367

[38] J. Lei, Z. Wang, J. Huo, S. Sang, C. Zhang, E. Zhu, T. Kong, F. Karada, J. Low, Y. Xiong, Visible light-driven acetaldehyde production from CO_2_ and H_2_O via synergistic vacancies and atomically dispersed Cu sites. Angew. Chem. Int. Ed. Engl. 64, e202422667 (2025). 39999322 10.1002/anie.202422667

[39] G. Kwon, Y. H. Choi, H. Lee, H. S. Kim, J. Jeong, K. Jeong, M. Baik, H. Kwon, J. Ahn, E. Lee, M. H. Cho, Interaction-and defect-free van der Waals contacts between metals and two-dimensional semiconductors. Nat. Electron. 5, 241–247 (2022).

[40] Y. Yao, Y. Zhu, C. Pan, C. Wang, S. Hu, W. Xiao, X. Chi, Y. Fang, J. Yang, H. Deng, S. Xiao, J. Li, Z. Luo, Y. Guo, Interfacial sp C-O-Mo hybridization originated high-current density hydrogen evolution. J. Am. Chem. Soc. 143, 8720–8730 (2021). 34100598 10.1021/jacs.1c02831

[41] H. Li, L. Zhu, J. Wang, L. Li, K. Shih, Development of nano-sulfide sorbent for efficient removal of elemental mercury from coal combustion fuel gas. Environ. Sci. Technol. 50, 9551–9557 (2016). 27508312 10.1021/acs.est.6b02115

[42] H. Yu, Y. Xue, B. Huang, L. Hui, C. Zhang, Y. Fang, Y. Liu, Y. Zhao, Y. Li, H. Liu, Y. Li, Ultrathin nanosheet of graphdiyne-supported palladium atom catalyst for efficient hydrogen production. iScience 11, 31–41 (2019). 30584958 10.1016/j.isci.2018.12.006 PMC6305764

[43] J. Li, L. Zhong, L. Tong, Y. Yu, Q. Liu, S. Zhnag, C. Yin, L. Qiao, S. Li, R. Si, J. Zhang, Atomic Pd on graphdiyne/graphene heterostructure as efficient catalyst for aromatic nitroreduction. Adv. Funct. Mater. 29, 1905423 (2019).

[44] Y. Fang, Y. Liu, L. Qi, Y. Xue, Y. Li, 2D graphdiyne: An emerging carbon material. Chem. Soc. Rev. 51, 2681–2709 (2022). 35253033 10.1039/d1cs00592h

[45] S. Chen, Y. Xue, Y. Gao, H. Wu, S. Chen, Y. Zheng, Y. Li, Interfacial atom rearrangement drives potential-adaptive electrocatalytic olefin hydrogenation. Angew. Chem. Int. Ed. Engl. 137, e202507269 (2025). 10.1002/anie.202507269 40297937

[46] X. Zheng, Y. Xue, H. Wu, J. Li, S. Chen, S. Chen, Y. Gao, Y. Li, Metal atom self-assemblies for conversion of CO_2_ to C_2_ Products. CCS Chem. 7, 3096–3106 (2025).

[47] Y. Xue, B. Huang, Y. Yi, Y. Guo, Z. Zuo, Y. Li, Z. Jia, H. Liu, Y. Li, Anchoring zero valence single atoms of nickel and iron on graphdiyne for hydrogen evolution. Nat. Commun. 9, 1460 (2018). 29654234 10.1038/s41467-018-03896-4 PMC5899097

[48] K. Song, X. Geng, H. Yin, Y. Shi, J. Wang, J. Yu, M. Bai, L. Wang, Y. Xue, C. Song, Y. Fan, Schottky engineering of GDYO@Pt to boost piezoelectric and oxidative stress modulation for accelerated cranial regeneration. Nat. Commun. 16, 8523 (2025). 41006255 10.1038/s41467-025-63550-8 PMC12475170

[49] G. Kresse, J. Furthmüller, Efficient iterative schemes for ab initio total-energy calculations using a plane-wave basis set. Phys. Rev. B 54, 11169–11186 (1996). 10.1103/physrevb.54.11169 9984901

[50] G. Kresse, J. Furthmüller, Efficiency of ab-initio total energy calculations for metals and semiconductors using a plane-wave basis set. Comput. Mater. Sci. 6, 15–50 (1996).

[51] J. P. Perdew, K. Burke, M. Ernzerhof, Generalized gradient approximation made simple. Phys. Rev. Lett. 77, 3865–3868 (1996). 10062328 10.1103/PhysRevLett.77.3865

[52] J. P. Perdew, J. A. Chevary, S. H. Vosko, A. K. Jackson, M. R. Pederson, D. J. Singh, C. Fiolhais, Atoms, molecules, solids, and surfaces: Applications of the generalized gradient approximation for exchange and correlation. Phys. Rev. B 46, 6671–6687 (1992). 10.1103/physrevb.46.6671 10002368

[53] S. Grimme, S. Ehrlich, L. Goerigk, Effect of the damping function in dispersion corrected density functional theory. J. Comput. Chem. 32, 1456–1465 (2011). 21370243 10.1002/jcc.21759

[54] S. Grimme, J. Antony, S. Ehrlich, H. Krieg, A consistent and accurate ab initio parametrization of density functional dispersion correction (DFT-D) for the 94 elements H-Pu. J. Chem. Phys. 132, 154104 (2010). 20423165 10.1063/1.3382344

[55] V. Wang, N. Xu, J.-C. Liu, G. Tang, W.-T. Geng, VASPKIT: A user-friendly interface facilitating high-throughput computing and analysis using VASP code. Comput. Phys. Commun. 267, 108033 (2021).

[56] J. V. Vondele, M. Krack, F. Mohamed, M. Parrinello, T. Chassaing, J. Hutter, Quickstep: Fast and accurate density functional calculations using a mixed Gaussian and plane waves approach. Comput. Phys. Commun. 167, 103–128 (2005).

