# Supplementary material for: Rational design of interfacial sp C─S─Zn hybridization in ZnS/graphdiyne for mercury vapor capture
Source: Sci Adv. 2026 Jun 19;12(25):eaef9068. doi: 10.1126/sciadv.aef9068 (PMC13281809; doi:10.1126/sciadv.aef9068)
Supplement: Supplementary file 1 — Supplementary Text Figs. S1 to S11 Tables S1 to S4 [file sciadv.aef9068_sm.pdf]

Science Advances
AAAS

Supplementary Materials for

Rational design of interfacial sp C─S─Zn hybridization in ZnS/graphdiyne for mercury vapor capture

Chuanqi Pan *et al.*

Corresponding author: Chuanqi Pan, panchuanqi@haut.edu.cn; Yuan Yao, yaoyuan231@ipe.ac.cn; Honghu Li, lhhsam@outlook.com

Sci. Adv. **12**, eaef9068 (2026)
DOI: 10.1126/sciadv.aef9068

This PDF file includes:

Supplementary Text
Figs. S1 to S11
Tables S1 to S4

Supplementary Text

Experimental details:

Materials

Zinc acetate dihydrate ($Zn(CH_3COO)_2 \cdot 2H_2O$, analytical reagent) were purchased from Tianjin Kemio Chemical Reagent Co., Ltd. Sodium sulfide nonahydrate ($Na_2S \cdot 9H_2O$, ≥98%) were purchased from Shanghai McLean Biochemical Technology Co., Ltd. Absolute ethanol (C_2H_5OH, analytical reagent) were purchased from China National Pharmaceutical Group Chemical Reagent Co., Ltd. Deionized water is obtained by the Ultra Pure Water Machine at the Youpu Laboratory.

Characterization

The characterization equipment is an X-ray photoelectron spectrometer (Thermo Fisher, ESCALAB 250Xi+, USA). The excitation source uses Al ka rays (HV=1486.6eV), and the charge correction is performed based on the C1s=284.80eV binding energy as the energy standard. Raman spectroscopy DXR (American Thermo Electron)with a laser excitation wavelength of 532 nm (parameters: accumulation time = 10 s, accumulations = 5, delay time = 0 s, ND filter = 10 %, laser = 532 nm, scanning range: 50-3600 cm^{-1}, power adjustable from 0-14 mw, spectral resolution: 1 cm^{-1}). Electron paramagnetic resonance spectrometer (Bruker A300). High resolution transmission electron microscope (FEI talos F200X, USA). Energy Dispersive Spectrometer (FEI super-X EDS). The specific surface areas of the samples were measured by nitrogen adsorption-desorption at 77 K on a Belsorp max instruments. Nitrogen sorption analysis was done with Belsorp-max instrument using the Brunauer-Emmet Teller method.

To detect the mercury species on the spent sample, the temperature programmed desorption of Hg (Hg-TPD) was conducted. The Hg-laden sample was firstly purged by N_2 (400 ml/min) at room temperature for 20 min. Then the sample was heated to 400 °C at a heating rate of 3 °C/min under N_2 atmosphere. Meanwhile, the desorbed mercury concentration was monitored by a mercury detecting device (QM201H, Suzhou Qing'an Instrument Co., Ltd).

Prior to each test, sampling of N_2 (background value) is performed to ensure that the mercury signal peak area of the mercury analyzer approaches zero. Mercury standard gas (with a known mercury concentration) is used to calibrate the analyzer, establishing a standard curve correlating mercury concentration with mercury signal peak area. The actual mercury concentration is linearly related to the mercury signal peak area. It can be seen that obtained Hg-TPD curve exhibits no baseline drift, indicating reliable test results. The mercury desorption amount (D_{Hg}) of the sample can be determined by integrating the mercury desorption curve. The desorption peak area of ZnS/GDY is larger, indicating a stronger adsorption capacity for mercury.

$$D_{Hg}=\int_0^t cq \ dt \tag{S1}$$

c: mercury concentration, q: gas flow rate

Fitting procedures for X-ray photoelectron spectroscopy (XPS)

The obtained XPS raw data were subjected to charge correction based on calibrating the binding energy of sp^2-C to 284.4 eV. A shirley background subtraction method was applied to establish the baseline and eliminate background signals from inelastically scattered electrons. For high-resolution C 1s XPS spectra, subpeaks corresponding to sp-C, sp^2-C, C-O, and C=O were sequentially introduced based on the peak positions reported in the literature. The area ratio of sp-C to sp^2-C was fixed to 2:1. Peak parameters were automatically optimized using the residual minimization algorithm within the software XPSpeak. The deconvolution results were compared with literature references to assess their validity. All the XPS spectrum data was subjected to charge compensation based on calibrating the binding energy of sp^2-C to 284.4 eV.

Performance evaluation

The Hg^0 removal performance of ZnS/GDY is evaluated by a lab-scale fixed bed reaction system, which is composed of gas feeding, quartz reactor, mercury detecting and tail gas treatment. Typically, about 70 mg sample is loaded into the quartz reactor equipped with a temperature control unit. Different gases with desired content including N_2, SO_2, etc. are provided from compressed gas cylinders and introduced into the feed gas with accurate flow control. Hg^0 vapor with desired concentration generated from a mercury permeation device is carried by N_2 and mixed thoroughly with other gas components before entering into the quartz reactor. The total flow rate is kept at 400 mL/min with N_2 as balancing gas. Before each performance evaluation experiment, the feed gas is switched to bypass and measured to achieve a stable inlet Hg^0 concentration with concentration fluctuation below 5%. Then the feed gas is diverted to pass through the sample and the Hg^0 concentration at outlet is monitored by a mercury detecting device (QM201H, Suzhou Qing'an Instrument Co., Ltd). The Hg^0 removal efficiency (E_{Hg}, %) is determined based om the inlet (Hg^0_{in}) and outlet (Hg^0_{out}) Hg^0 concentrations and Hg^0 adsorption capacity (q_t) can be calculated as follows:

$$E_{Hg}=\left(1-\frac{Hg^0{}_{out}}{Hg^0{}_{in}}\right)\times 100\% \tag{S2}$$

$$q_t=\frac{1}{m}\int_0^t (Hg^0_{in}-Hg^0_{out})\times F\times dt\times 10^{-3} \tag{S3}$$

where Hg^0_{in} and Hg^0_{out} represent the inlet and outlet Hg^0 concentrations, respectively, μg/m^3; m represents the sample mass, g; F represents the gas flow rate, m^3/min; t is the time for reaction, min; q_t represents the Hg^0 adsorption capacity, mg/g.

Calculation details

Density functional theory (DFT) was employed to carry out first-principles calculations by using the Vienna ab-initio simulation package (VASP). In structural relaxation, exchange and correlation effects were calculated via the

Perdew-Burke-Ernzerhof functional (GGA-PBE). The self-consistency accuracy was set as 10^{-6} eV and the force convergence was set as 0.02 eV·Å^{-1}. And the plane wave energy cutoff was set as 400 eV. K-points were sampled in the Monhorst-Pack grid for the first Brillouin zone integration. The vacuum layer with 15 Å was set to prevent mirror interactions. All atoms were relaxed in the structural relaxation and simulations were dispersion-corrected by DFT-D3 to compensate for the calculation error of weak interactions. The VASPKIT software (version 1.5.1) was employed to analyze wave function files. All molecular dynamics simulation was performed under the canonical (NVT) ensemble of Nose-Hoover thermostats with time step set to be 1 fs. Charge transfers were calculated and the differential charge density was analyzed using the Bader charge method. As for the adsorption configurations of small molecules, the adsorption energy (E_{ad}) was defined as:

$$E_{ad} = E_{adsorbate+substrate} - (E_{adsorbate} + E_{substrate}) \quad (S4)$$

where $E_{adsorbate}$, $E_{substrate}$, *and* $E_{adsorbate+substrate}$ are the total energies of the free adsorbate, the corresponding support, and the support with the adsorbate in the same slab, respectively. All three types of energies were derived from the scf calculations using the same calculated setting parameters. With this definition, a negative value indicates an exothermic adsorption process, meaning the final state is energetically more stable than the isolated components.

Ab initio molecular dynamics (AIMD) calculations were carried out using the CP2K code. All calculations employed a mixed Gaussian and planewave basis sets. The generalized gradient approximation exchange-correlation functional of Perdew, Burke, and Enzerhof (PBE) was used. The valence electron wavefunction was expanded in a double-zeta basis set with an energy cutoff of 300 Ry. To compensate the long-range van der Waals dispersion interaction between the adsorbate and the framework, the DFT-D3 scheme with an empirical damped potential term was added into the energies obtained from exchange-correlation functional in all calculations. The NVT ensemble was applied for the graphdiyne systems. Number of steps was 30 ps and the step size was 1 fs.

Fig. S1.

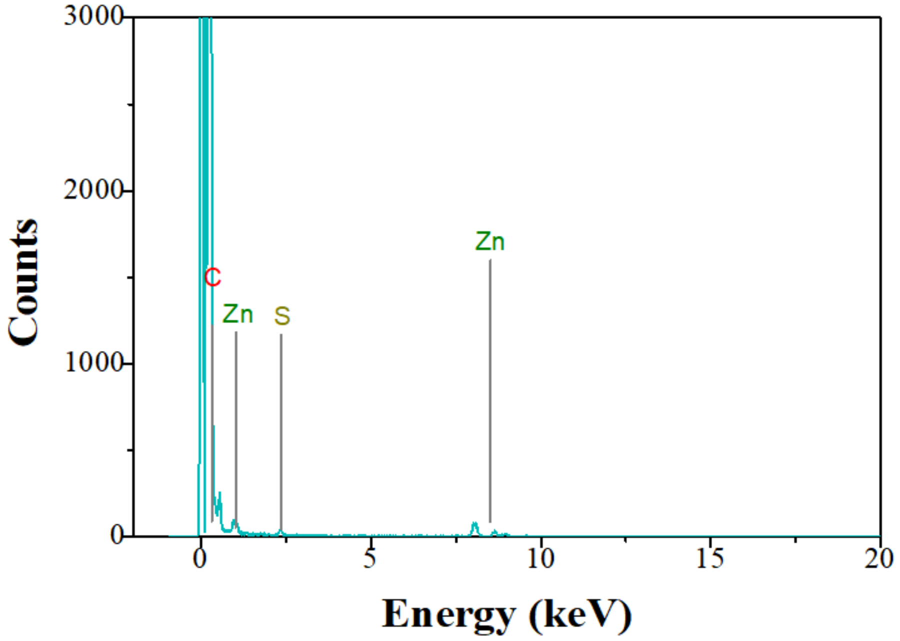

Figure S1. The EDS spectra of ZnS/GDY. The EDS spectra of ZnS/GDY obtained from the Fig 1E.

Fig. S2.

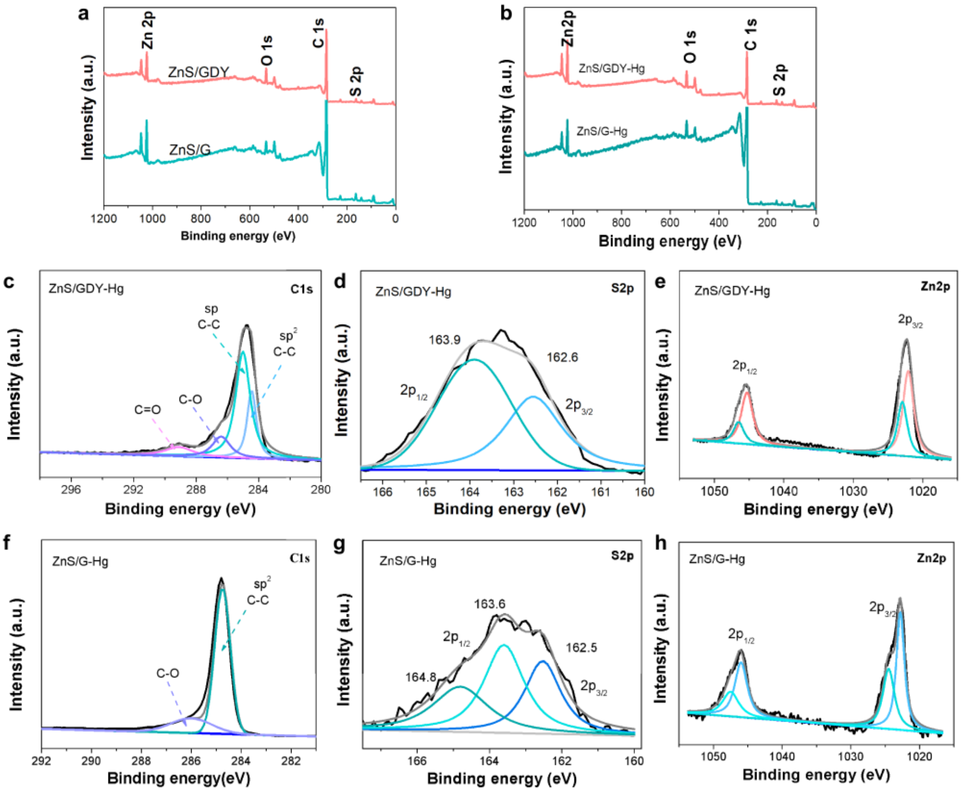

Figure S2. XPS characterization testing before and after performance evaluation of all samples. (**A**) The XPS survey spectra of ZnS/GDY and ZnS/G. (**B**) The XPS survey spectra of ZnS/GDY and ZnS/G after after Hg adsorption. The high-resolution (**C**) C 1s, (**D**) S 2p, (**E**) Zn 2p of ZnS/GDY after Hg adsorption.The high-resolution (**F**) C 1s, (**G**) S 2p, (**H**) Zn 2p of ZnS/G after Hg adsorption.

Fig. S3.

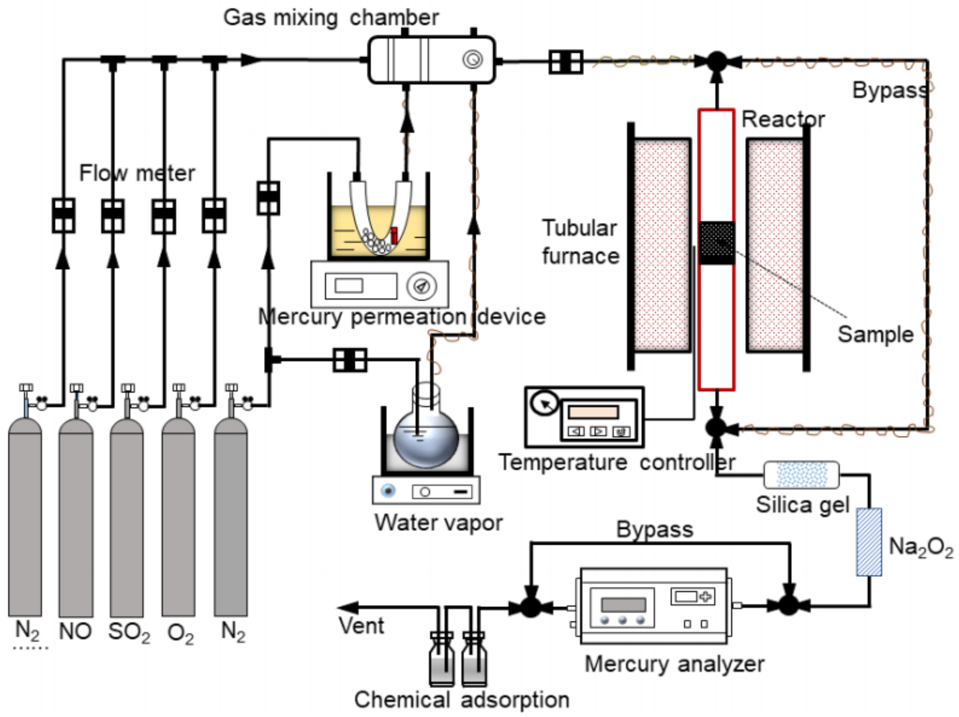

Figure S3. Schematic diagram of performance evaluation device. Schematic diagram of the lab-scale fixed-bed reaction system (RightsLink license from Elsevier Ltd.).

Fig.S4.

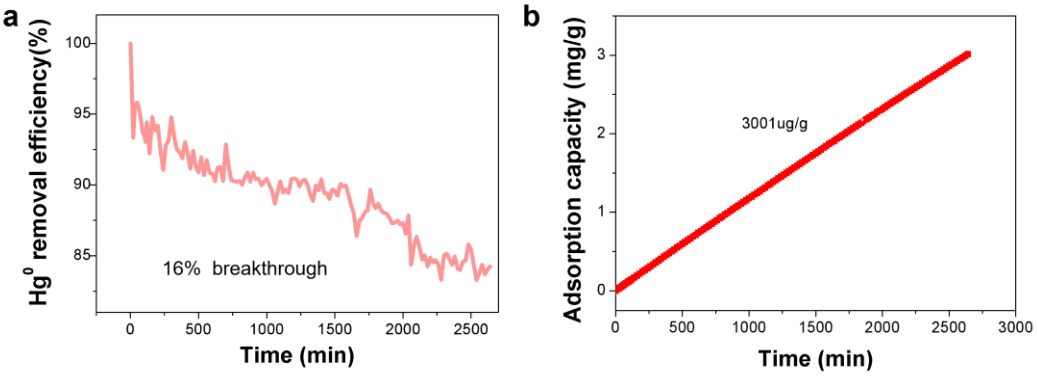

Figure S4. The breakthrough curve testing and adsorption energy evaluation. (**A**) The breakthrough curve and (**B**) The Hg adsorption capacity of ZnS/GDY.

Fig. S5.

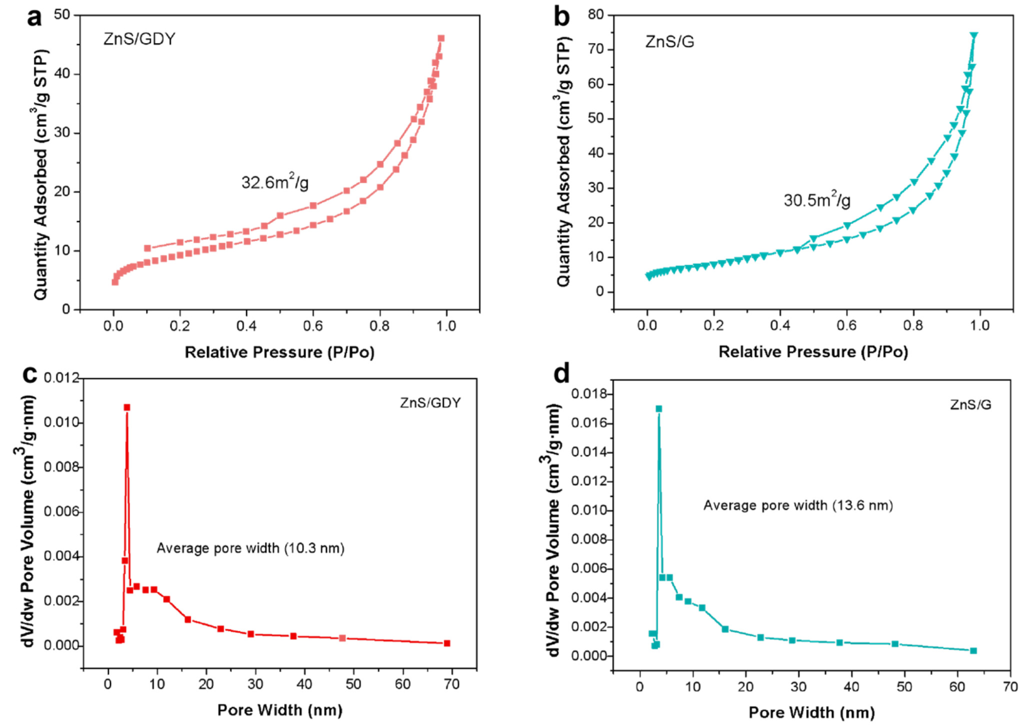

Figure S5. The analysis of specific surface area and pore structure. (**A**) The N_2 adsorption/desorption curves of ZnS/GDY. (**B**)The N_2 adsorption/desorption curves of ZnS/G. (**C**) The pore distribution curves of ZnS/GDY. (**D**)The pore distribution curves of ZnS/G.

Fig.S6.

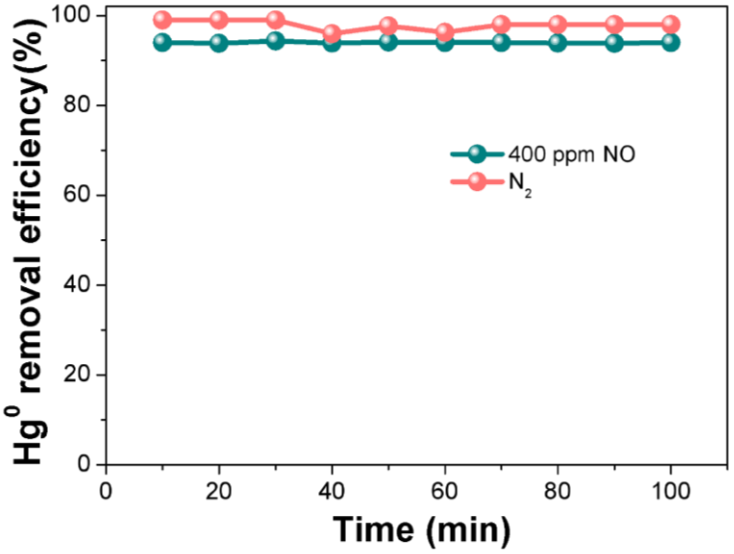

Figure S6. The evaluation of anti NO molecular interference performance. The Hg^0 adsorption performances of ZnS/GDY under different working conditions Hg^0+N_2 and Hg^0+NO.

Fig.S7.

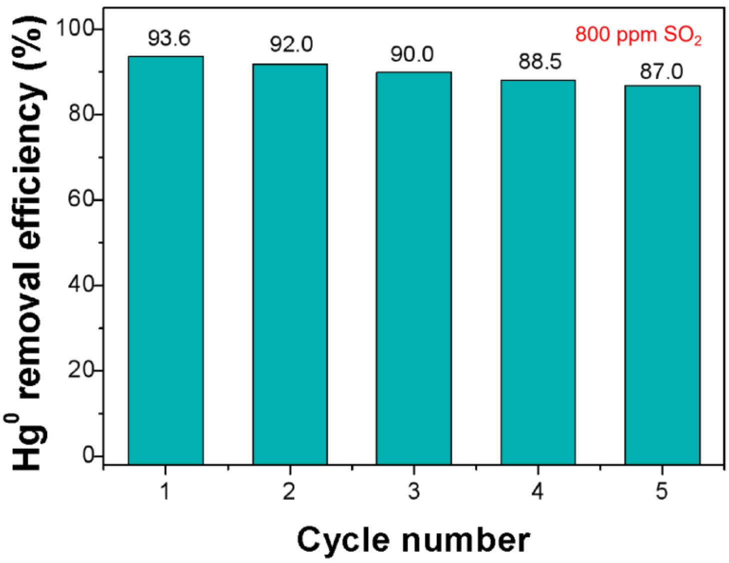

Figure S7. The cycle regeneration performance evaluation. The performance of ZnS/GDY during 5 adsorption-desorption cycles.

Fig. S8.

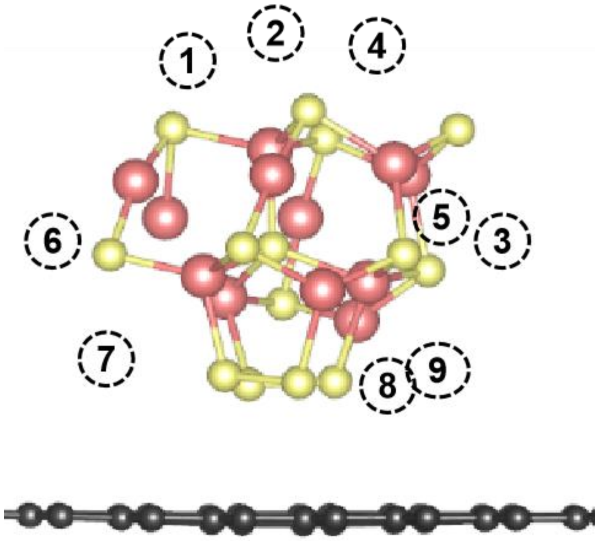

Figure S8. The calculation of the adsorption of Hg atoms by ZnS/GDY at different sites. Side views of the geometric structures for different adsorption sites of the Hg on ZnS/G (The yellow, pink and black spheres refer to S, Zn and C atoms, respectively).

Fig. S9.

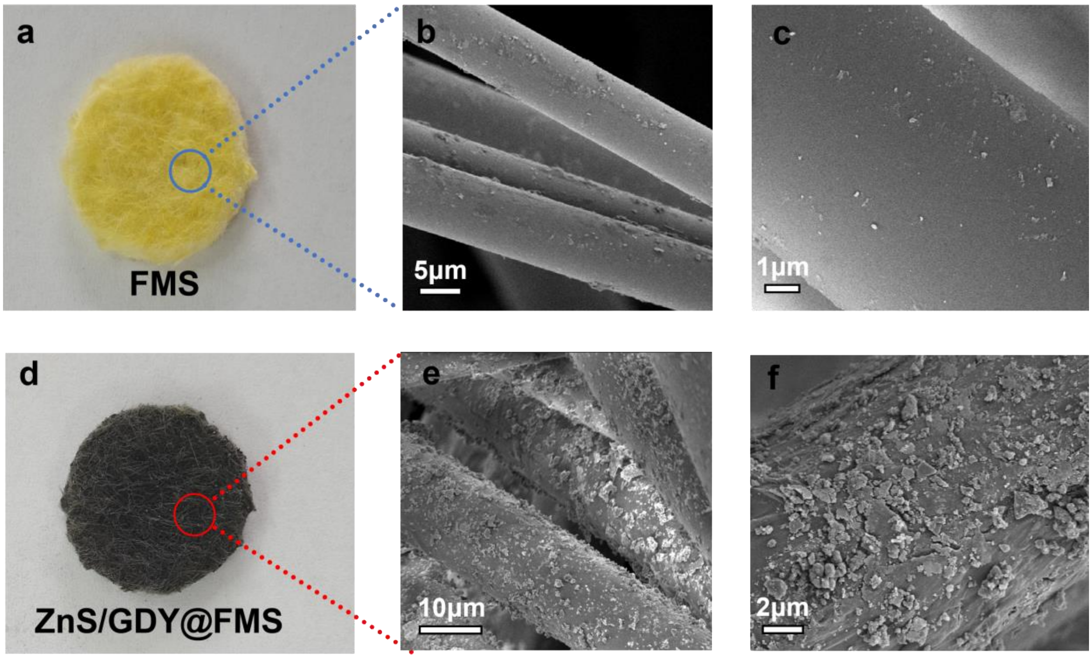

Figure S9. The SEM images of bare FMS and ZnS/GDY@FMS. (**A**) The digital photograph of bare FMS (Same image as in Fig.5B). (**B**) and (**C**) The SEM images of bare FMS at different magnifications. (**D**) The digital photograph of ZnS/GDY@FMS (Same image as in Fig.5B). (**E**) and (**F**) The SEM images of ZnS/GDY@FMS at different magnifications.

Fig. S10.

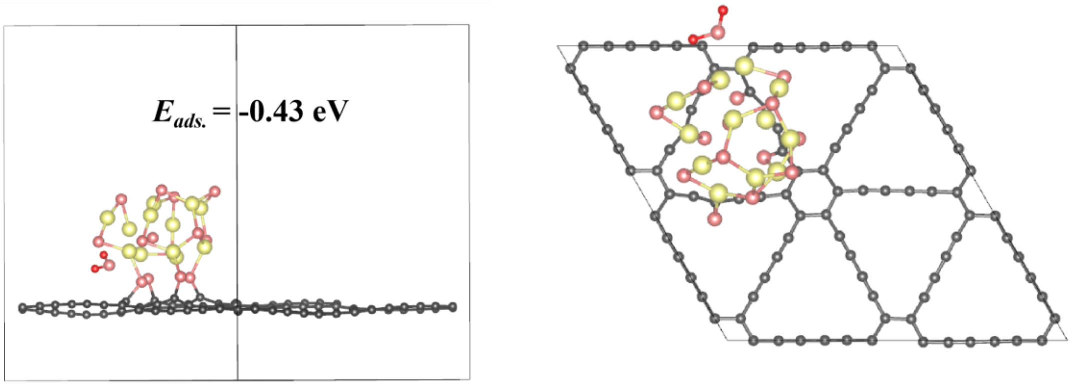

Figure S10. The calculation of the adsorption of SO_2 by ZnS/GDY. Side view and Top view of the geometric structures for the SO_2 on ZnS/G. (The yellow, pink, black and red spheres refer to S, Zn, C and O atoms, respectively).

Fig. S11.

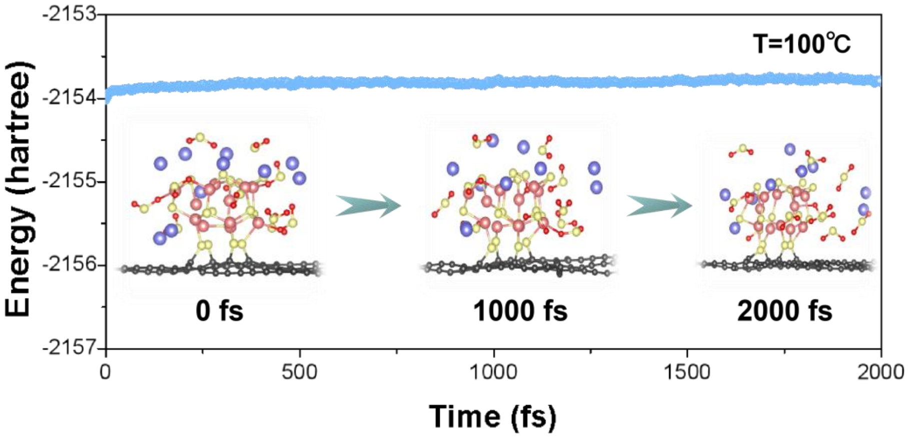

Figure S11. The molecular dynamics simulation of the effect of reaction temperature (100℃) on the stability of ZnS/GDY with SO_2 +Hg molecules. The molecular dynamics simulation of the effect of reaction temperature (100℃) on the stability of ZnS/GDY with SO_2 +Hg molecules. The whole reaction process is uncovered by displaying the snapshots of critical structures at 0 fs, 1000 fs and 2000 fs in order.

Table S1.

Supplementary Table S1. The quantitative results of the EDS spectra obtained from the Fig 1E.

Element	wt.%	at.%
C	99.03	99.76
Zn	0.30	0.115
S	0.67	0.124

Table S2.

Supplementary Table S2. The summary of adsorption configuration and adsorption energies of Hg atoms at different sites on the graphdiyne.

Adsorption configuration	Site	Adsorption energy (eV)
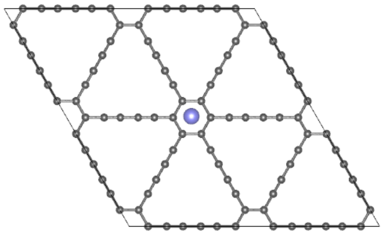	Site 1	-0.19
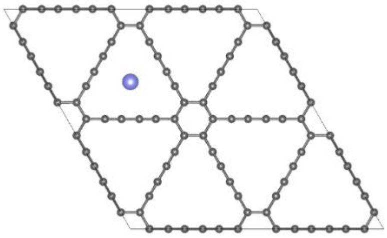	Site 2	-0.24
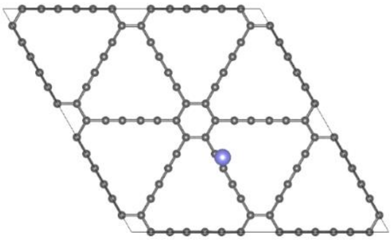	Site 3	-0.15
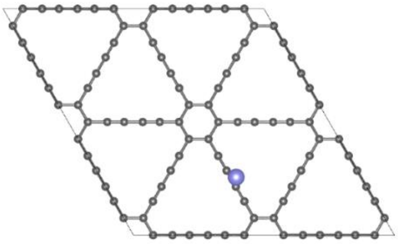	Site 4	-0.14

Table S3.

Supplementary Table S3. The summary of adsorption configuration and adsorption energies of Hg atoms at different sites on the ZnS/GDY.

Adsorption configuration	Site	Adsorption energy (eV)
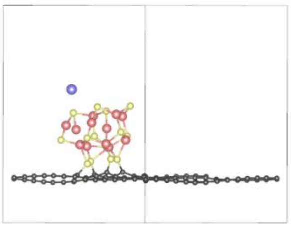	Site 1	-0.19
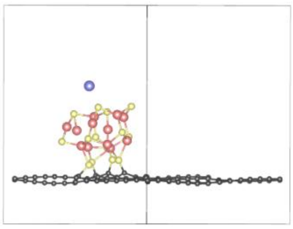	Site 2	-0.21
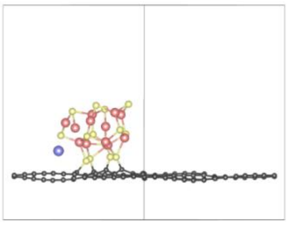	Site 3	-1.06
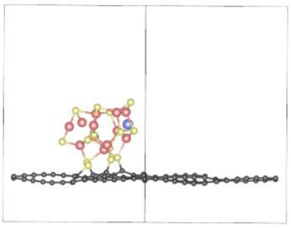	Site 4	-0.28
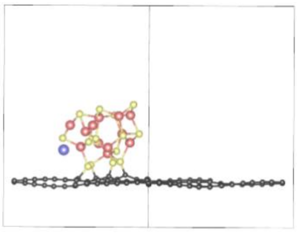	Site 5	-1.16
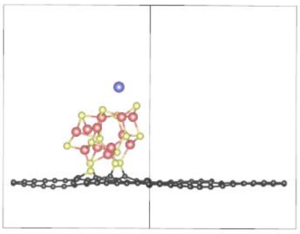	Site 6	-1.30

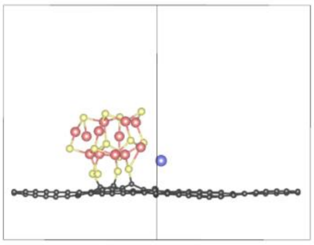	Site 7	-1.38
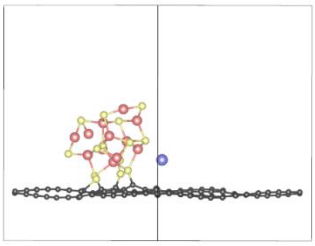	Site 8	-1.50
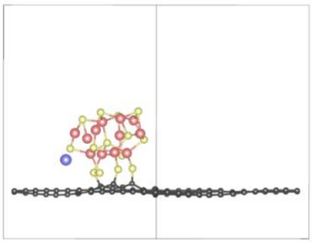	Site 9	-1.79

Table S4.

Supplementary Table S4. The summary of adsorption configuration and adsorption energies of Hg atoms at different sites on the ZnS/G.

Adsorption configuration	Site	Adsorption energy (eV)
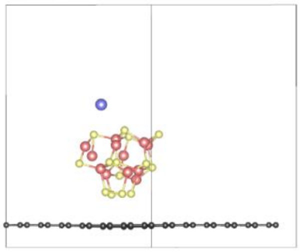	Site 1	-0.19
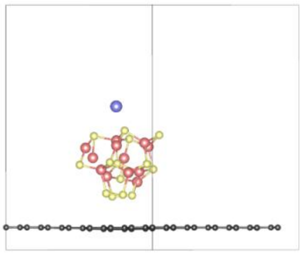	Site 2	-0.24
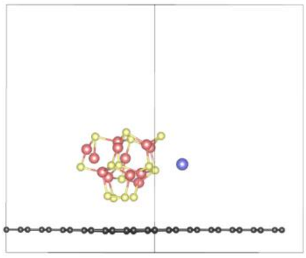	Site 3	-0.27
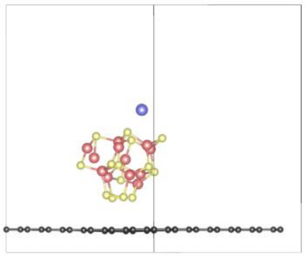	Site 4	-0.28
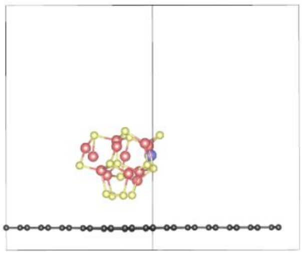	Site 5	-0.34

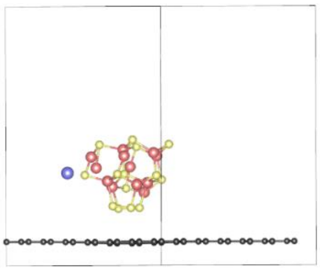	Site 6	-0.59
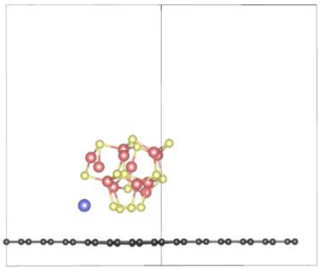	Site 7	-0.40
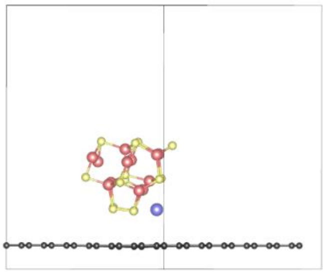	Site 8	-0.59
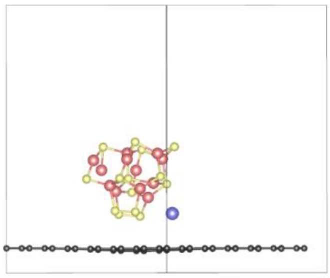	Site 9	-0.60
